# Customised and Noncustomised Birth Weight Centiles and Prediction of Stillbirth and Infant Mortality and Morbidity: A Cohort Study of 979,912 Term Singleton Pregnancies in Scotland

**DOI:** 10.1371/journal.pmed.1002228

**Published:** 2017-01-31

**Authors:** Stamatina Iliodromiti, Daniel F. Mackay, Gordon C. S. Smith, Jill P. Pell, Naveed Sattar, Debbie A. Lawlor, Scott M. Nelson

**Affiliations:** 1 School of Medicine, University of Glasgow, Glasgow Royal Infirmary, Glasgow, United Kingdom; 2 Institute of Health and Wellbeing, University of Glasgow, Glasgow, United Kingdom; 3 Department of Obstetrics and Gynaecology, University of Cambridge, Rosie Hospital, Cambridge, United Kingdom; 4 NIHR Cambridge Biomedical Research Centre, Cambridge, United Kingdom; 5 Institute of Cardiovascular and Medical Sciences, University of Glasgow, Glasgow, United Kingdom; 6 MRC Integrative Epidemiology Unit at the University of Bristol, Bristol, United Kingdom; University of Manchester, UNITED KINGDOM

## Abstract

**Background:**

There is limited evidence to support the use of customised centile charts to identify those at risk of stillbirth and infant death at term. We sought to determine birth weight thresholds at which mortality and morbidity increased and the predictive ability of noncustomised (accounting for gestational age and sex) and partially customised centiles (additionally accounting for maternal height and parity) to identify fetuses at risk.

**Methods:**

This is a population-based linkage study of 979,912 term singleton pregnancies in Scotland, United Kingdom, between 1992 and 2010. The main exposures were noncustomised and partially customised birth weight centiles. The primary outcomes were infant death, stillbirth, overall mortality (infant and stillbirth), Apgar score <7 at 5 min, and admission to the neonatal unit. Optimal thresholds that predicted outcomes for both non- and partially customised birth weight centiles were calculated. Prediction of mortality between non- and partially customised birth weight centiles was compared using area under the receiver operator characteristic curve (AUROC) and net reclassification index (NRI).

**Findings:**

Birth weight ≤25th centile was associated with higher risk for all mortality and morbidity outcomes. For stillbirth, low Apgar score, and neonatal unit admission, risk also increased from the 85th centile. Similar patterns and magnitude of associations were observed for both non- and partially customised birth weight centiles. Partially customised birth weight centiles did not improve the discrimination of mortality (AUROC 0.61 [95%CI 0.60, 0.62]) compared with noncustomised birth weight centiles (AUROC 0.62 [95%CI 0.60, 0.63]) and slightly underperformed in reclassifying pregnancies to different risk categories for both fatal and non-fatal adverse outcomes (NRI -0.027 [95% CI -0.039, -0.016], *p* < 0.001). We were unable to fully customise centile charts because we lacked data on maternal weight and ethnicity. Additional analyses in an independent UK cohort (*n* = 10,515) suggested that lack of data on ethnicity in this population (in which national statistics show 98% are white British) and maternal weight would have misclassified ~15% of the large-for-gestation fetuses.

**Conclusions:**

At term, birth weight remains strongly associated with the risk of stillbirth and infant death and neonatal morbidity. Partial customisation does not improve prediction performance. Consideration of early term delivery or closer surveillance for those with a predicted birth weight ≤25th or ≥85th centile may reduce adverse outcomes. Replication of the analysis with fully customised centiles accounting for ethnicity is warranted.

## Abstract 2

In an analysis of 979,912 term singleton pregnancies, Stamatina Iliodromiti and colleagues compare using customised and noncustomised birth weight centiles for prediction of stillbirth and infant mortality and morbidity.

## Author Summary

**Why Was This Study Done?:**

- In developed countries, one-third of stillbirths and infant deaths occur at term.
- There are multiple clinical definitions at term of what constitutes a small- or large-for-gestation fetus, with <10th centile and >90th centile commonly used. Whether these statistical thresholds can accurately identify fetuses at risk of mortality or morbidity is unknown.
- Customised birth weight centiles (accounting for sex, gestation, and maternal characteristics) are increasingly being adopted by many maternity units. However, whether they can identify term fetuses at risk of death more accurately than noncustomised centiles is unknown.

**What Did The Researchers Do And Find?:**

- We examined data on 979,912 term singleton pregnancies over a 19-y period in Scotland. With external validation of our findings on an independent UK cohort (*n* = 10,515).
- We studied the associations of birth weight centiles (noncustomised and partially customised) with stillbirth, infant mortality, admission to the neonatal unit and Apgar score <7 at 5 min. In addition, we assessed whether partially customised centiles perform better in predicting adverse outcomes compared with noncustomised centiles. We were unable to assess fully customised centiles as we did not have data on maternal ethnicity and weight.
- We found that birth weight ≤25th or ≥85th centile (both partially and noncustomised) are associated with greater risk of adverse outcomes. Partially customised centiles did not identify more fetuses at risk of death compared with noncustomised centiles.

**What Do These Findings Mean?:**

- Adverse outcomes frequently occur in term fetuses. Closer surveillance or earlier delivery of those fetuses with a predicted birth weight ≤25th or ≥85th centile may reduce adverse outcomes.
- Replication of the analysis with fully customised birth weight centiles is required.

## Introduction

Infants who are born at the extremes of birth weight have a higher risk of adverse perinatal outcome [1]. In developed countries, one-third of stillbirths and infant deaths occur at term [2], yet no consensus exists about what defines a small or large fetus or infant at term. A variety of methods have been used, including absolute birth weight (most commonly <2,500 g and >4,000 g or 4,500 g), or statistical thresholds outside the expected birth weight for gestational age (commonly <10th or >90th centile or, for more severe phenotypes, two standard deviations) [3–7]. Whether these thresholds optimally define the risk of perinatal mortality and morbidity at term is unknown. Furthermore, some advocate that birth weight percentiles should account for maternal characteristics known to be associated with fetal growth, such as weight, height, parity, and ethnicity. However, there is conflicting evidence whether customised charts perform better than noncustomised centiles in predicting adverse perinatal outcome [8–11] and the strength of evidence for supporting this approach, particularly for term infants, has been challenged [12,13].

The aims of this study were (1) to determine the shapes and magnitudes of the associations between birth weight centile and infant death, stillbirth, infant mortality and stillbirth combined, Apgar score <7 at 5 min, and admission to the neonatal unit and (2) to compare the accuracy of predicting mortality (infant mortality and stillbirth) using noncustomised (accounting for infant sex and gestational age at birth) birth weight centile charts versus partially customised (additionally accounting for maternal height and parity) birth weight centile charts.

## Methods

The Privacy Advisory Committee of the Information Services Division (ISD) of the National Services Scotland awarded ethical approval for access to and linkage of the datasets (www.isdscotland.org). All data were nonidentifiable, and individual informed consent from participants was not required.

This study is reported as per Strengthening the Reporting of Observational Studies in Epidemiology (STROBE) guidelines (S1 Checklist).

### Study Population

We linked four Scotland-wide databases: the Scottish Morbidity Record 02 (SMR02), the National Records of Scotland (NRS), the Scottish Stillbirth and Infant Death Survey (SSBIDS), and the General Registrar for Scotland’s death certificate database, to provide comprehensive obstetric, neonatal, infant, and mortality data for all women discharged from Scottish maternity hospitals. Details of the datasets and the quality assurance procedures are provided in Supporting Information (full lists of data collected are available here: http://www.adls.ac.uk/nhs-scotland/maternity-inpatient-and-day-case-smr02/?detail). The data can be accessed from the National Services Scotland (www.isdscotland.org) following approval.

### Inclusion and Exclusion Criteria

We obtained SMR02 data on all infants delivered in Scotland between 1 January 1992 and 31 December 2010 inclusive, the latter equating to the most recent data available at the time of data extraction. Our analyses were restricted to singleton births, in women aged over 10 y, with a gestational age at delivery between 37 and 43 wk inclusive. We excluded infant deaths due to congenital anomalies or isoimmunisation and stillbirths due to congenital abnormalities. Death ascribed to congenital anomaly was defined as any structural or genetic defect incompatible with life or potentially treatable but causing death.

### Outcomes, Exposures, and Potential Modifying Variables

The primary outcomes were infant mortality (deaths up to 1 y of age), stillbirths (intrapartum or antepartum deaths), combined stillbirth and infant mortality, low Apgar score (<7) at 5 mins, and admission to neonatal unit (special care or neonatal intensive care unit).

The exposures were noncustomised birth weight centiles (sex and gestation specific) and partially customised birth weight centiles (sex and gestation specific and adjusted for maternal height and parity) [14]. Transformation of data to partially customised centiles was performed with the GROW centiles bulk calculator: http://www.gestation.net/GROW_documentation.pdf. Customised centile charts typically also adjust for maternal weight and ethnicity. However, this information was not available in the routine data sources employed. White British ethnicity was assumed for all pregnancies since only 2% of babies were born to women in ethnic minority groups (ISD personal communication) in Scotland over this period. Weight was assumed to be 66 Kg for all women (the default of the GROW bulk calculator and the median of UK women at the start of pregnancy). Given the lack of adjustment for maternal weight and ethnicity, we refer to the adjusted percentiles as "partially customised." Noncustomised percentiles were internally standardized for gestational age (in weeks) and sex. Gestational age has been confirmed by ultrasound in the first half of pregnancy in more than 95% of women in the UK since the early 1990s [15].

We considered that year of delivery, parity, and diabetes (either existing or gestational) might be effect modifiers of the relationships between birth weight and outcomes. Data on year of delivery and parity were obtained from SMR02. The ICD codes recorded on the SMR02 record were used to ascertain gestational diabetes: 6488 (ICD9) O244, O249 (ICD10) and pre-existing diabetes: 250, 6480 (ICD9) E10-14, O240-1, O243 (ICD10). Any associations between birth weight and fatal outcomes might differ in those who die after delivery as a result of delivery complications (anoxia, trauma, or intracranial haemorrhage [ICH]). We, therefore, repeated all main analyses with deaths related to these causes only.

In addition to the procedures and audits used to ensure high-quality data on mortality, we explored the face validity of these data further by assessing associations of known risk factors, such as a previous history of infant/fetal mortality or morbidity and smoking.

### Statistical Analysis

On being granted data access, all authors agreed on the statistical analysis plan prior to commencing the main analysis. The analysis of the ALPSAC cohort was undertaken in response to comments from peer reviewers. All analyses were performed using Stata (version 13, StataCorp LP, College Station, Texas) and R version 3.2.3 (R Foundation for Statistical Computing, Vienna, Austria).

#### Main analyses

We used multivariable cubic regression splines to model the associations between birth weight centiles (non- and partially customised) and each outcome using the mvrs command in Stata. The mvrs command uses a closed-test approach to select the most appropriate spline model from a number of competing spline models [16]. Briefly, this involves estimating a model using a cubic spline for the continuous variable allowing for a pre-determined amount of nonlinearity at specific quintiles, or knots, of the continuous variable resulting in a model with a default 4 degrees of freedom. If this “flexible” model is a significantly better fit than a model which omits the continuous predictor, then the testing procedure moves on to compare the “flexible model” with a linear model. If these two models are not significantly different to each other, as measured by the change in deviance, then the more parsimonious linear model, i.e., a straight line, is chosen. If the change in deviance is significant then the testing procedure continues by comparing a spline with 3 degrees of freedom to a spline with the lowest degrees of freedom and again testing for a significant change in deviance. The process continues until the change in deviance between the models is no longer significant. The spline with the fewest degrees of freedom is then chosen as the most appropriate model. For present analyses, we selected the position of the knots in the spline model as being where the association of the birth weight centile with an outcome changed (in direction or magnitude). Once knots had been identified, we then used binomial with a log link regression to estimate the relative risks (RR) of each outcome comparing birth weight below and above the chosen centile thresholds (knots), referent to the birth weight centiles within the thresholds. We decided a priori that if our initial analyses identified centile thresholds that differed from those that are used currently in clinical practice (i.e. 10th and 90th centiles, respectively, to define small-for-gestational-age [SGA] and large-for-gestational-age [LGA] births) we would determine the RR of each outcome comparing birth weight <10th and >90th centile referent to birth weights between 10th to 90th centile, using binomial-logit regression. Based on these thresholds, we estimated the additional number of pregnancies that would need pre-emptive delivery in order to prevent one fatal event, assuming that earlier delivery would result in 69% reduction in the risk of mortality [17], compared with the traditional birth weight thresholds of 10th and 90th centiles. We repeated the same analysis for partially customised birth weight centiles thresholds. To compare the performance of non- with partially customised birth weight centiles at predicting fatal outcomes (infant deaths and stillborn), we compared the area under the curve of receiver operator characteristics (AUROC) and the net reclassification index (NRI) for each model. All formulae are provided in the online Supporting Information.

#### Exploring effect modification

Differences in associations of birth weight with mortality (stillbirth or infant) by parity, year of delivery, and diabetes were examined by presenting stratified results—two categories of parity (nulliparous and multiparous), four categories for year of delivery (1992–1996, 1997–2001, 2002–2006, 2007–2010), and two categories for diabetes (yes, no)—and by testing for statistical interaction between these categories and birth weight in their association with outcomes, using a likelihood ratio test.

#### Dealing with missing data

Complete data were available on over 99% of the cohort for all variables included in the main analyses, with the exception of maternal height (Table 1). Maternal height was missing on 19% of participants. We imputed maternal height using all baseline maternal characteristics, previous obstetric history, and obstetric outcomes in the index pregnancy. We generated five imputed datasets using the chained equations approach based on each conditional density of a variable given other variables [18]. We did not impute other variables because of the low rate of missing values.

**Table 1:** Maternal and offspring characteristics stratified by pregnancy outcomes.

Characteristics	Stillbirths	Infant deaths	Survived to 1 y of Age	*p*-Value
***N* (%)**	1,672 (0.2)	1,093 (0.1)	977,147 (99.7)	
**Maternal Age (years)**	29.0 (24.0 to 34.0)	27.0 (22.0 to 32.0)	29.0 (24.0 to 33.0)	0.0001
Missing *N* (%)	1 (0.06)	0	10 (0.001)	
**Maternal Height (cm)**	162.8 ± 6.5	162.2 ± 6.7	163.0 ± 6.5	0.002
Missing *N* (%)	342 (20.5)	261 (23.9)	182,312 (18.7)	
**Parity (*n*, %)**				< 0.0001
Nulliparous	855 (51.4)	480 (44.0)	441,120 (45.3)	
Multiparous	809 (48.6)	611 (56.0)	531,719 (54.7)	
Missing *N* (%)	8 (0.5)	2 (0.2)	4,308 (0.4)	
**Year of delivery (*n*)**				0.03
1992–1996	533	284	279,196	
1997–2001	402	352	250,075	
2002–2006	388	266	238,440	
2007–2010	349	191	209,436	
Missing (%)	0	0	0	
**Pre-existing Diabetes *N* (%)**	24 (1.4)	6 (0.6)	3,226 (0.3)	< 0.0001
Missing *N* (%)	0	0	0	
**Gestational Diabetes *N* (%)**	10 (0.6)	6 (0.6)	5,009 (0.5)	0.88
Missing *N* (%)	0	0	0	
**Gestational Age (weeks)**	39.0 (38.0 to 40.0)	40.0 (38.0 to 40.0)	40.0 (39.0 to 41.0)	0.0001
Missing *N* (%)	0	0	0	
**Birth weight (g)**	3,120 (2,692 to 3,560)	3,203 (2,892 to 3,610)	3,460 (3,140 to 3,780)	0.0001
Missing *N* (%)	0	0	0	
**Male Sex *N* (%)**	872 (52.2)	582 (53.3)	498,067 (51.0)	0.2
Missing *N* (%)	1 (0.06)	0	29 (0.003)	

Data are presented in median (interquartile range) or mean ± standard deviation (SD), unless indicated otherwise.

Percentages are expressed as the % of the nonmissing values. (NND: neonatal death, SIMD: Scottish Index of Multiple Deprivation)

#### Assessing the impact of not having data on maternal ethnicity and weight

We undertook additional analyses in an independent UK Cohort—the Avon Longitudinal Study of Parents and Children (ALSPAC), which has data on all of the variables required for customisation and an ethnic distribution similar to that of the main Scottish population used here, with 98% being of white British origin [19,20]. We used the Bulk calculator (as in our main study) to define fully customised centile charts for all participants (*n* = 10,378 following exclusions similar to the analyses completed in the main study) and then recalculated these first with all weight values removed (so that each woman would be given the default 66 Kg) and then additionally with the 2% who were not white British changed to white British. We calculated the proportions of those defined as SGA, normal for gestational age and LGA birth weight, with missing weight and ethnicity data to the gold standard of full-customisation, using both the 10th and 90th centiles and 25th and 85th centiles to define SGA and LGA, respectively.

## Results

### Cohort Characteristics

**Fig 1:**
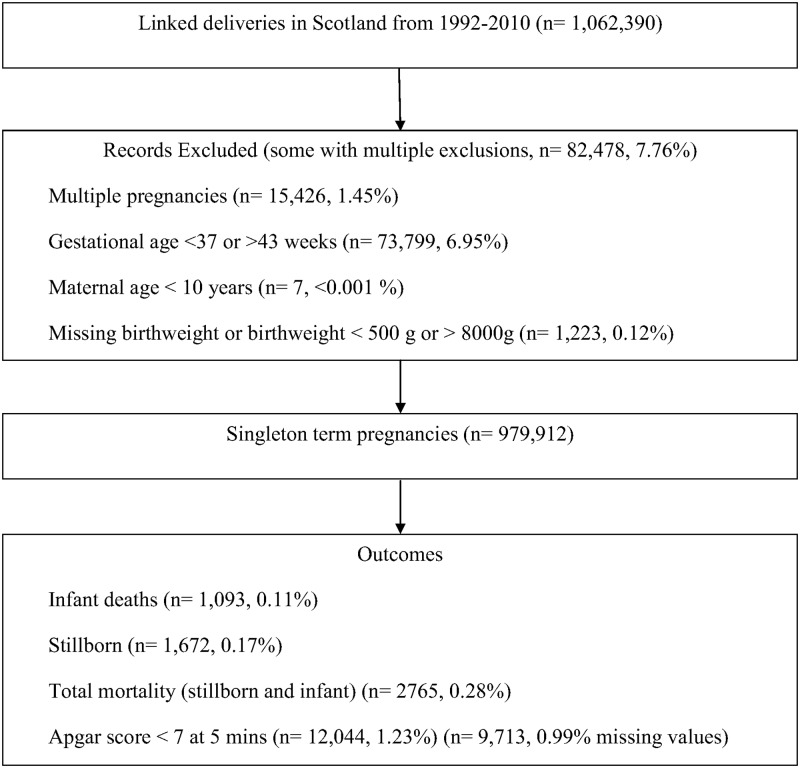
Definition of eligible cohort and analysis sample.

Between 1 January 1992 and 31 December 2010, there were 1,062,390 deliveries in Scotland. After pre-specified exclusions, the analysis cohort included 979,912 term deliveries (Fig 1). Among these deliveries 2,765 (0.3%) resulted in death: 1,672 (0.2%) were stillborn and 1,093 (0.1%) suffered infant death. There were 12,044 (1.2%) neonates who had an Apgar score <7 at 5 mins and 62,217 (6.4%) were admitted to the neonatal unit. Table 1 shows the maternal and offspring characteristics used in our main analyses by mortality outcomes (stillbirth and infant deaths compared to infants alive at 1 y) and S1 Table shows additional characteristics used to validate the mortality data by these categories. Women who delivered a stillborn were more likely to be nulliparous, smoke, live in deprived areas, and have pre-existing diabetes. Women who delivered neonates who died within their first year of life were more likely to be younger, live in more deprived areas, smoke, and have a history of previous stillbirth or neonatal death. Stillborn infants and infant deaths had lower birth weight at the time of delivery compared with neonates who survived beyond 1 y of age. S2 Table lists the causes of infant deaths.

### Adverse Outcomes in Relation to Birth Weight Centile

**Fig 2:**
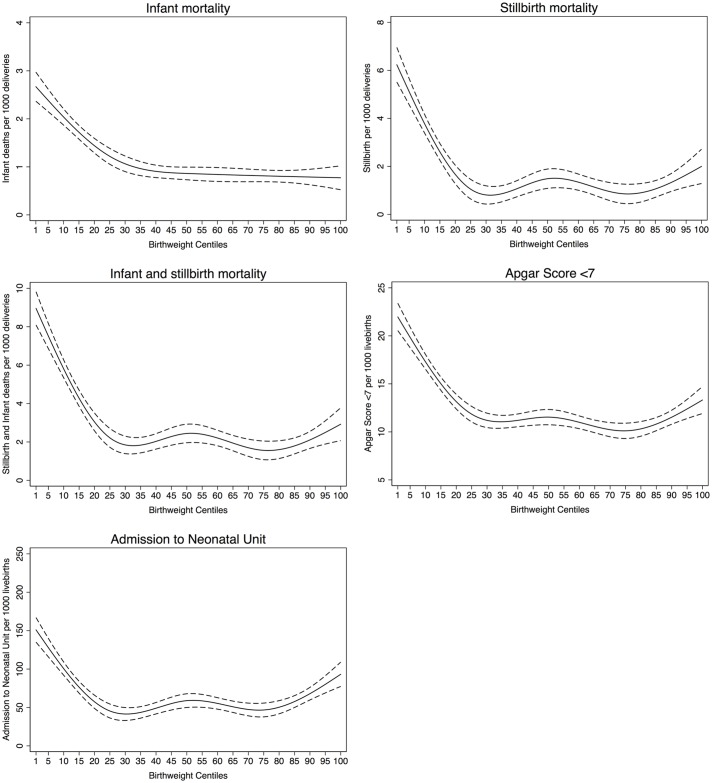
Mortality and morbidity outcomes relative to noncustomised birth weight centiles.

For all five outcomes, the patterns of risk were similar but the magnitude of risk was greater for stillbirth and overall mortality. The risk started to increase at approximately the 25th centile of noncustomised birth weight centiles and increased monotonically from that point (Fig 2). For stillbirth, low Apgar score and admission to the neonatal unit there was also some evidence of risk increasing from approximately the 85th centile. Infant mortality risk remained low in all participants who were above the 25th centile. Similar patterns and position of knots were observed for partially customised birth weight centiles for all five outcomes (S1 Fig). When we restricted the analyses to infant mortality attributed to anoxia, trauma, or intracranial haemorrhage the results were similar, though the magnitude of associations were somewhat weaker, for both noncustomised and partially customised birth weight centiles (S2 Fig). S3 and S4 Figs show that knot points and associations were similar in different categories of parity and year of delivery. However, diabetes (existing or gestational) seems to modify the associations between birth weight and fatal events (*p* for interaction < 0.001) and fatal events increase with increasing birth weight centile (linear association) in infants of mothers with diabetes, whereas in those without diabetes the 25th and 85th centiles knot points were still evident (S5 Fig).

Table 2 shows the risk was similar for all five outcomes for those with a birth weight ≤25th centile or ≥85th centile referent to birth weight between 25th to 85th centiles for both noncustomised and partially customised centiles. In addition, we present the risk of all five outcomes for birth weight based on the traditional thresholds (<10th centile and >90th centile) referent to birth weights between 10th and 90th centiles. The risk of all mortality and morbidity outcomes was only slightly stronger for these more restrictive thresholds than the 25th and 85th centiles inferred by our data.

**Table 2:** Relative risk (RR) with 95% confidence intervals of adverse outcomes for chosen and traditional birth weight centiles (noncustomised and customised) referent to the birth weight centiles included between the thresholds.

	Birth weight centiles	RR of Infant deaths	RR of Stillbirth	RR of Combined mortality	RR of low Apgar score at 5 mins (<7)	RR of admission to Neonatal Unit
Noncustomised centiles	**< 10th**	2.50 (2.16, 2.90)	3.96 (3.55, 4.41)	3.33 (3.05, 3.63)	1.74 (1.66, 1.83)	2.35 (2.31, 2.40)
	**> 90th**	0.78 (0.62, 0.99)	1.21 (1.02, 1.43)	1.02 (0.89, 1.17)	1.08 (1.01, 1.14)	1.45 (1.42, 1.49)
	**≤ 25th**	2.03 (1.79, 2.31)	2.73 (2.46, 3.02)	2.42 (2.24, 2.62)	1.44 (1.38, 1.50)	1.64 (1.61, 1.67)
	**≥ 85th**	0.92 (0.76, 1.12)	1.20 (1.03, 1.40)	1.08 (0.96, 1.22)	1.06 (1.01, 1.12)	1.36 (1.33, 1.39)
Customised centiles	**< 10th**	2.57 (2.23, 2.96)	3.93 (3.53, 4.37)	3.34 (3.07, 3.64)	1.61 (1.54, 1.70)	2.27 (2.23, 2.31)
	**> 90th**	0.93 (0.75, 1.14)	1.29 (1.10, 1.51)	1.13 (1.00, 1.28)	1.10 (1.04, 1.16)	1.55 (1.52, 1.59)
	**≤ 25th**	2.14 (1.89, 2.43)	2.59 (2.34, 2.88)	2.40 (2.22, 2.60)	1.41 (1.35, 1.46)	1.61 (1.58, 1.64)
	**≥ 85th**	0.98 (0.81, 1.18)	1.25 (1.07, 1.45)	1.13 (1.01, 1.27)	1.11 (1.05, 1.16)	1.48 (1.45, 1.51)

Combined mortality is the summation of infant deaths and stillborn. (Referent population for thresholds of 10th and 90th centile is birth weight centiles between 10th to 90th and for thresholds of 25th and 85th centiles is birth weight centiles between 25th to 90th centiles.)

### Prediction of Mortality by Noncustomised or Partially Customised Birth Weight Centile

Discrimination of prediction of mortality was not improved using a partially customised birth weight centile (AUROC 0.61 [0.60 to 0.62]), compared with a noncustomised birth weight centile (AUROC model 2 0.62 [0.60 to 0.63]; *p* < 0.0001) (S3 Table). The model with partially customised birth weight centiles as the predictor of overall mortality also slightly underperformed in reclassifying pregnancies to different risk categories (for both pregnancies with a fatal and nonfatal event) compared with the model using noncustomised birth weight centiles as the exposure (NRI -0.027 [95% CI -0.039, -0.016], *p* < 0.001) (Table 3).

**Table 3:** Reclassification of fatal and nonfatal events (stillbirth or infant death).

Model with noncustomised birth weight centiles (adjusted for gestational week and sex)	Model with customised birth weight centiles (new model)			
Frequency (%)	< 0.18	0.18–0.26	> 0.26	*Total*
***Fatal Events***				
**< 0.18**	429	62	0	*491*
**0.18–0.26**	50	364	57	*471*
**> 0.26**	1	80	1722	*1*,*803*
***Total***	*480*	*506*	*1*,*779*	*2*,*765*
***Nonfatal events***				
**< 0.18**	195,426	36,107	26	*231*,*559*
**0.18–0.26**	21,517	194,238	37,675	*253*,*430*
**> 0.26**	6	29,754	462,398	*492*,*158*
***Total***	*216*,*949*	*260*,*099*	*500*,*099*	*977*,*147*

Net Reclassification Index (NRI) for Events = P(up/events) -P(down/events) = [(62+0+57)-(50+1+80)]/2,765 = -0.0043 or -0.43%

NRI for non-events = P(down/non-events) -P(up/non-events) = [(21,517+6+29,754)–(36,107+26+37,675)]/977,147 = -0.0231 or -2.31%

Overall NRI (95% confidence intervals) = -0.027 (-0.039, -0.016), *p* < 0.001

### Clinical Utility of Novel Thresholds—Numbers Needed to Treat

Expediting delivery for an anticipated birth weight of ≤25th noncustomised centile rather than <10th (assuming that the intervention would have 69% effectiveness for mortality), would result in pre-emptive delivery of 159,025 additional women to prevent 377 fatal events (stillbirth and infant deaths). Hence, to prevent one death, 422 (95% CI 381 to 468) additional pregnancies below this new threshold would require to be delivered. If the equivalent ≤25th threshold was used for the partially customised centiles, intervention in an additional 463 (95% CI 417 to 516) pregnancies would be required to prevent one fatal event. Adopting the threshold of ≥85th centile for defining large for gestation babies rather than >90th centile and delivering them earlier, assuming 69% effectiveness of intervention, would require an additional 721 (95% CI 598 to 947) or 826 (95% CI 638 to 1,091) pre-emptive deliveries, respectively for noncustomised and partially customised centiles, to prevent one fatal event.

### Additional Analyses Exploring the Impact of Customisation for Maternal Ethnicity and Weight

In the ALSPAC cohort partially customised centile charts that did not adjust for maternal weight accurately identified SGA in comparison with fully customised charts, with 97% of those identified as SGA with full-customisation being identified with partial (weight removed) customisation when either the 10th (S4 Table) or 25th (S5 Table) thresholds were used. Accuracy for LGA was somewhat poorer with 84% and 85% being correctly identified, with the 90th and 85th percentiles, respectively (S4 and S5 Tables). With additional change of all ethnicities to white the results did not differ from those shown (for removal of weight only) in S4 and S5 Tables.

## Discussion

Being born too small or too large is associated with an increased risk of mortality and morbidity [1,5,7]. Despite dramatic improvements in maternal and neonatal care, we showed that even at term (37 to 43 gestational weeks) birth weight remains strongly associated with the risk of stillbirth and infant death, low Apgar score, and admission to the neonatal unit. An increased risk of mortality and morbidity was evident at term with birth weights ≤25th and ≥85th centile irrespective of whether noncustomised or partially customised centiles were used, with similar associations observed for potentially preventable infant deaths due to anoxia, trauma, or intracranial haemorrhage. These thresholds may not apply to diabetic pregnancies, in which there is evidence of increasing mortality with greater birth weight. Given that partially customised centiles exhibited weaker associations with mortality than simpler noncustomised centiles, their increasingly wide adoption by health care providers [21] for identifying may not be appropriate for assessing risks of adverse perinatal outcome at term.

Prior to term, fetal biometry is used to detect abnormal growth trajectories, with the overall clinical management of ongoing fetal health and timing and mode of delivery guided by evidence-based guidelines [10]. This is particularly the case for preterm small-for-gestational-age (SGA) fetuses. There is less emphasis and guidance on the management of the term fetus, yet this group contributes one-third of all stillbirths and infant deaths and associated substantial neonatal morbidity. A previous population analysis suggested that there was a progressive increase in perinatal mortality from 37 wk gestation and elective induction of labour at term was associated with decreased odds of perinatal death without an associated increase in emergency caesarean section delivery [22]. There has been no widespread adoption of this policy despite randomised controlled trials also suggesting induction of labour does not increase operative delivery [23,24]. This is largely due to concerns regarding increasing caesarean section rates and that induction of labour is associated with an increased risk of neonatal admission to a special care unit [22]. Our analysis suggests that guidelines on using biometry to predict birth weight in term infants and considering early term delivery for those anticipated to have a birth weight ≤25th centile for gestation week at term may reduce overall mortality while minimising iatrogenic neonatal morbidity [25,26]. Consideration of all available information (e.g., growth trajectory) and additional testing (e.g., umbilical and uterine Doppler) may facilitate differentiation between the pathological and nonpathological cases, with interventions focusing on those identified as pathological [6]. Of importance, delivery at 37–38 wk is associated with the lowest perinatal risk of death [26]. While the DIGITAT study did not show a difference in a composite measure of adverse neonatal outcome between early term induction of labour and expectant management in those with suspected fetal growth restriction (below the 10th centile), the authors suggested intense monitoring of those who are keen on expectant management to minimise the risk of stillbirth and neonatal morbidity [27]. We have previously shown that early term delivery (37–38 wk) is associated with a small increase in special educational needs compared with delivery at 40–41 wk [28]. However, this study was in a general population and not those selected on the basis of evidence that fetal growth may be faltering, hence, it is unclear whether earlier intervention in those whose fetus is predicted to be faltering grow will lead to greater risk of long-term educational outcomes. Interestingly, the same study showed a progressive increase in the risk of special educational needs with decreasing birth weight below the 20th percentile. For infants with a birth weight <3^rd^ percentile, the risk was doubled. Hence, interventions focused on improved care of poorly grown fetuses could potentially have longer term benefits in childhood.

Our results also support considering earlier delivery of those fetuses ≥85th centile at term, with a recent randomised controlled trial suggesting that induction of labour at 37–38^+6^ wk for those in the >95th centile reduces shoulder dystocia [29]. But there are no clear guidelines for preventing macrosomia and large-for-gestational-age (LGA), which is becoming increasingly common due to the increasing prevalence of obesity among women of reproductive age [30]. Randomised trials of effects on stillbirth or infant mortality are unlikely to be conducted because of the extremely large sample size that would be required and therefore for these rare but extremely important outcomes large population level data like ours are required to guide practice. For anticipated birth weights ≤25th or ≥85th centile, early delivery would be anticipated to reduce mortality and delivery related morbidity, as delaying delivery to more than 39 wk in at-risk infants has previously been shown to increase the number of stillbirths and infants affected by macrosomia [31].

The clinical utility of routine second and third trimester ultrasound for the detection of fetuses with abnormal growth and at increased risk of neonatal complications has recently been reported [6]. An additional benefit of these ultrasounds would be an estimate of birth weight using internationally validated growth charts [3], which could be adopted into a prediction model at 36 wk to guide care at term if comprehensive induction of labour for estimated birth weight beyond ≤25th or ≥85th centiles was to be avoided [3,4].

The process of customisation of birth weight centiles has an additional major difference to the conventional approach, over and above accounting for maternal characteristics. The growth reference used is the distribution of weight at a given gestational age based on ultrasonic estimation of fetal weight, rather than actual birth weight. This is important preterm, as previous studies have shown that preterm birth is associated with fetal growth restriction [32]. A consequence is that use of customised centiles markedly increases the proportion of preterm births which are defined as SGA. As preterm birth is one of the major determinants of perinatal morbidity and mortality, this may explain some of the apparent strengthening of associations following customisation described in previous studies [14]. At term, this effect is less dominant and the relative risk of stillbirth is similar irrespective of whether actual birth weight, customised or an intrauterine reference ranges used [33]. That partially customised centiles exhibit poorer performance in prediction of mortality suggests that the maternal characteristics that they adjust for are not purely physiological and may be associated with pathological impairment of fetal growth resulting in inappropriate misclassification of high-risk term fetuses. Maternal short stature, primiparity, and ethnicity are all independently associated with perinatal mortality, supporting their pathological contribution to fetal growth [34,35]. We were only able to partially customise birth weight charts as we did not have data on maternal ethnicity or weight. However, additional analyses of the independent ALSPAC cohort suggested that in a similar population (i.e., in which the vast majority are white), lack of complete ethnicity data would not have biased our results. Similarly, lack of maternal weight appeared to have very little impact on defining SGA, although the extent of misclassification was somewhat higher for LGA, but the majority of LGA (over 84%) were still correctly classified. Consistent with the lack of major bias due to potentially misclassifying ethnicity in 2% of our study, a recent multicentre international study (*n* = 20,486) indicated that ethnicity had a minimal effect on fetal growth in healthy women [3], though another smaller study (*n* = 1,737) suggested that Hispanic, Asian, and black ethnicities are associated with differential growth and up to 245 g lower median estimated fetal weight than white Europeans by 39 wk gestation [36]. However, very few women of reproductive age in Scotland are from these ethnic groups. Maternal height was imputed for 19%, but the distribution of height in the imputed database was the same as women with measured height and when we repeated analyses restricted to those women with complete data on height and all other variables the results were essentially unchanged from those presented here using imputed data (data available from authors on request).

Our study included around one million deliveries. We included all eligible deliveries in Scotland over a 19-y period, thereby avoiding selection bias. We linked four national datasets to maximise data completeness. The routine data sources are subject to quality assurance checks and perform well in terms of completeness and accuracy. The detail provided by these datasets allowed us to examine a range of different outcomes and we used robust methods to calculate the RR of the outcomes of infant mortality, stillbirth, Apgar score <7 at 5 min, and neonatal admission for birth weight at term. Measurement of birth weight was not subject to quality control measures, but represents routine clinical practice, and is not subject to the error that can occur in research assessments of birth weight taking place within the first few days (rather than immediately as in clinical practice) because of the large decrease in weight observed during the first two days of life [37]. We were unable to examine specific nonfatal complications but assumed that substantive neonatal morbidity would be associated with neonatal unit admission. We examined a range of relative risks to identify thresholds, and different populations may wish to accept lower or higher risks of mortality and morbidity to guide thresholds for intervention. In particular, we cannot assume that the thresholds that we have identified here would be appropriate for other (than white European) populations. We acknowledge that using birth weight as a proxy of estimated fetal weight (EFW) may have inflated some of the associations. However, the difference between EFW and actual birth weight has been quoted up to an average of 10% [38] and we have previously shown that universal ultrasonography performs well as a screening test for SGA (under the 10th birth weight centile) with an AUROC of ~0.9 [6]. However, a key area for future research is to identify methods which help discriminate healthy SGA and LGA infants from infants which are SGA or LGA as a consequence of pathological processes. Combinations of tests (e.g., ultrasound and biomarkers) may reduce the false positive rate and allow targeting of interventions to pregnancies with high absolute risks of adverse outcome, and this is an active area of current research.

In conclusion, term fetuses remain at substantive risk of infant death, stillbirth and neonatal morbidity. Birth weight identifies those at greatest risk, and our results support consideration of early delivery, increased surveillance or additional testing for those with an anticipated birth weight ≤25th centile or ≥85th centile (rather than the widely used 10th and 90th centiles, respectively) proposed to reduce adverse outcomes. Replication of our results in other independent large datasets is warranted. A clinical trial looking at morbidity outcomes (since the small number of mortality outcomes renders a clinical trial with primary outcome stillbirth and infant deaths practically unfeasible) will clarify whether early term intervention at the proposed thresholds of birth weight will be beneficial. In Scotland, and similar countries, there is no evidence that partially customised charts perform better at identifying those term infants at risk than noncustomised charts.

## Supporting Information

S1 Fig Noncustomised and partially customised birth weight centiles and mortality and morbidity outcomes. (TIF)

S2 Fig Noncustomised and partially customised birth weight centiles for infant death due to anoxia, trauma, or ICH. (TIF)

S3 Fig Noncustomised and partially customised birth weight centiles for mortality for nulliparous and parous women. (TIF)

S4 Fig Noncustomised and partially customised birth weight centiles for mortality for year of delivery. (TIF)

S5 Fig Noncustomised and partially customised birth weight centiles for mortality in nondiabetic and diabetic pregnancies. (TIF)

S1 PRISMA Checklist PRISMA Checklist. (DOC)

S1 Table Additional characteristics of the cohort in the original and the imputed datasets. (DOCX)

S2 Table Causes of Infant Deaths at term (*n* = 1,093). (DOCX)

S3 Table Prediction of stillbirth or infant mortality using customised birth weight or gestation and sex specific birth weight centiles in imputed data. (DOCX)

S4 Table Categorisation into small, normal and large for gestational age comparing partial-customisation to the gold-standard of full-customisation using 10th and 90th centile thresholds to define SGA and LGA. Analyses undertaken in the ALSPAC cohort (*n* = 10,378).
(DOCX)

S5 Table Categorisation into small, normal, and large for gestational age comparing partial customisation to the gold standard of full customisation using 25th and 85th centile thresholds to define SGA and LGA. Analyses undertaken in the ALSPAC cohort (*n* = 10,378).
(DOCX)

S1 Text Supporting text. (DOCX)

## Abbreviations

ALSPAC: Avon Longitudinal Study of Parents and Children
AUROC: area under the receiver operator characteristic curve
EFW: estimated fetal weight
ICH: intracranial haemorrhage
LGA: large for gestational age
NRI: net reclassification index
RR: relative risks
SGA: small for gestational age
