# Supplementary figures and images for: Customised and Noncustomised Birth Weight Centiles and Prediction of Stillbirth and Infant Mortality and Morbidity: A Cohort Study of 979,912 Term Singleton Pregnancies in Scotland
Source: PLoS Med. 2017 Jan 31;14(1):e1002228. doi: 10.1371/journal.pmed.1002228 (PMC5283655; doi:10.1371/journal.pmed.1002228)

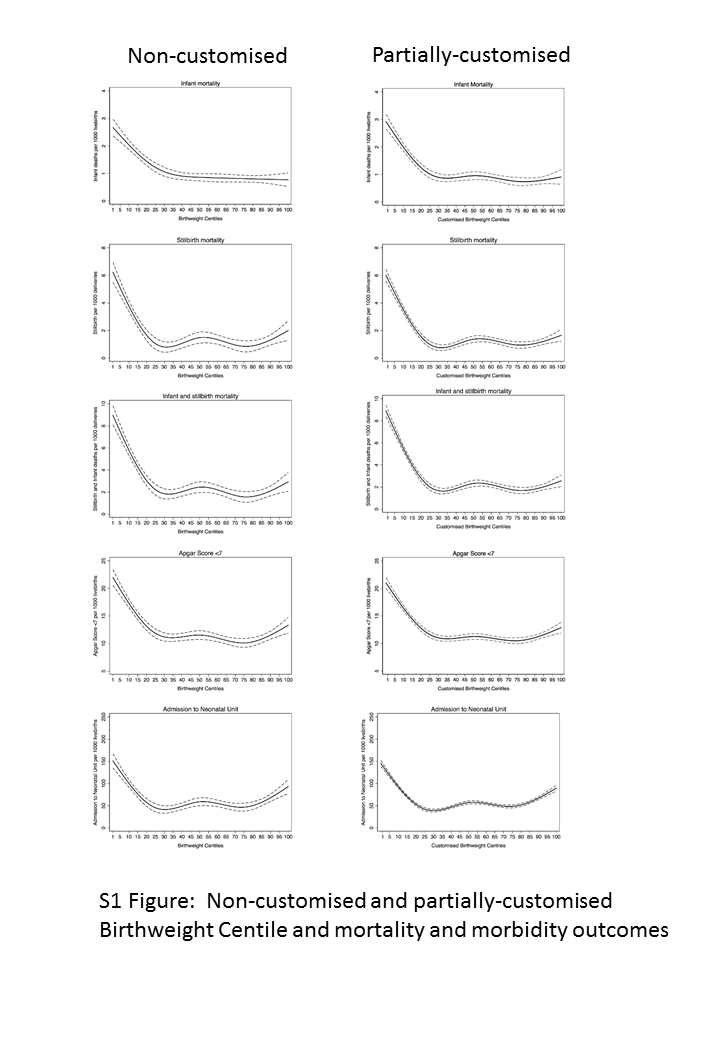

Supplement: S1 Fig — (TIF) [file pmed.1002228.s001.tif]

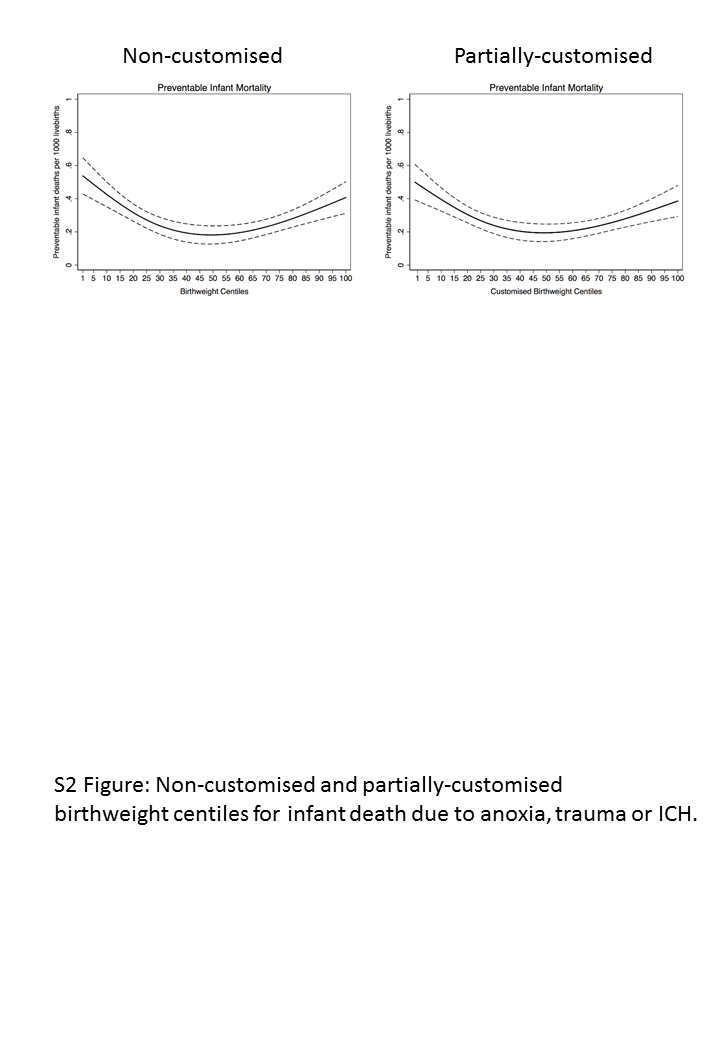

Supplement: S2 Fig — (TIF) [file pmed.1002228.s002.tif]

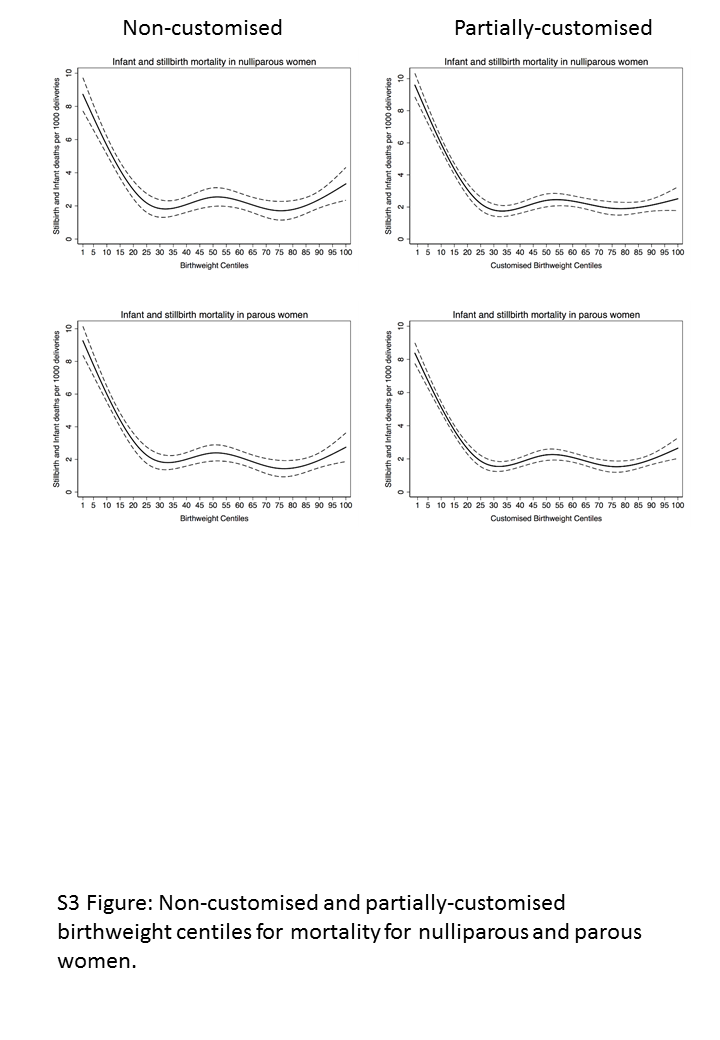

Supplement: S3 Fig — (TIF) [file pmed.1002228.s003.tif]

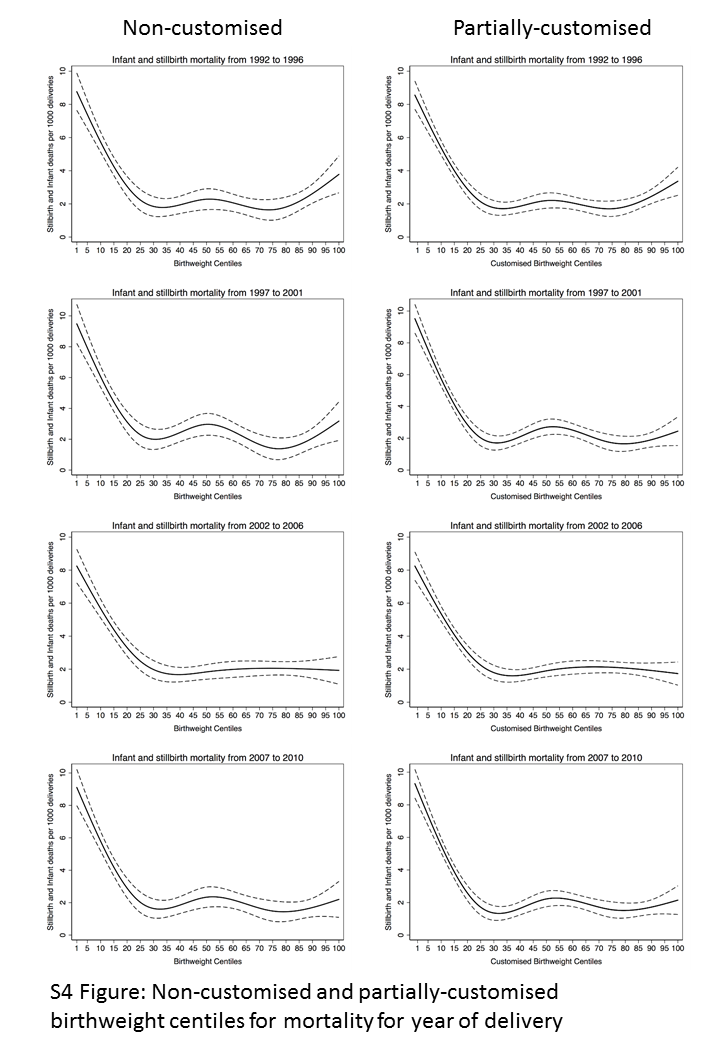

Supplement: S4 Fig — (TIF) [file pmed.1002228.s004.tif]

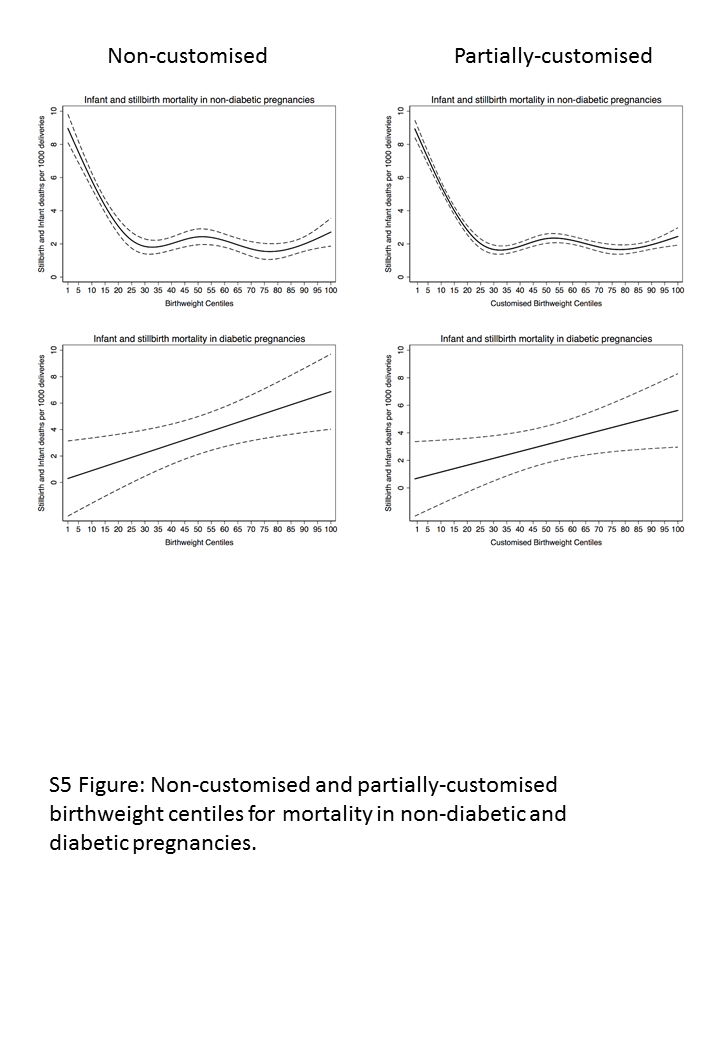

Supplement: S5 Fig — (TIF) [file pmed.1002228.s005.tif]
