# Supplementary material for: Customised and Noncustomised Birth Weight Centiles and Prediction of Stillbirth and Infant Mortality and Morbidity: A Cohort Study of 979,912 Term Singleton Pregnancies in Scotland
Source: PLoS Med. 2017 Jan 31;14(1):e1002228. doi: 10.1371/journal.pmed.1002228 (PMC5283655; doi:10.1371/journal.pmed.1002228)
Supplement: S1 Table — (DOCX) [file pmed.1002228.s007.docx]

**S1 Table:** Further characteristics of the cohort in the original and the imputed datasets.

| **Characteristics** | **Stillbirths** | **Infant deaths** | **Survived to 1 year of Age** | **P value** |
| --- | --- | --- | --- | --- |
| History of NND (n, %)  Missing N (%) | 4 (0.3)  259 (15.5) | 13 (1.4)  169 (15.5) | 3263 (0.4)  164,184 (16.8) | < 0.0001 |
| History of Stillbirth (n, %)  Missing N (%) | 39 (0.03)  259 (15.5) | 7 (0.8)  166 (15.2) | 4,589 (0.6)  164,145 (16.8) | < 0.0001 |
| History of Miscarriage (n, %)  Missing N (%) | 359 (21.5)  1 (0.06) | 233 (21.3)  0 | 199,206 (20.4)  272 (0.03) | 0.41 |
| History of Termination of Pregnancy (n, %)  Missing N (%) | 189 (11.3)  1 (0.06) | 110 (10.1)  0 | 100,222 (10.3)  260 (0.03) | 0.3 |

| SIMD N (%)  1-2 (most deprived)  3-4  5-6  7-8  9-10 (least deprived)  Missing N (%) | 498 (30.0)  334 (20.1)  317 (19.1)  272 (17.1)  229 (13.7)  10 (0.6) | 407 (37.3)  240 (22.0)  187 (17.1)  134 (12.3)  123 (11.3)  2 (0.2) | 253,034 (26.0)  200,157 (20.5)  178,876 (18.4)  171,089 (17.6)  170,277 (17.5)  3,714 (0.4) | < 0.0001 |
| --- | --- | --- | --- | --- |
| Married N (%)  Missing N (%) | 738 (57.2)  381 (22.8) | 395 (48.8)  283 (25.9) | 468,092 (62.8)  232,292 (23.8) | < 0.0001 |
| Smoking N (%)  Current Smoker  Missing N (%) | 513 (34.7)  193 (11.5) | 455 (47.2)  128 (11.7) | 237,721 (26.9)  92,438 (9.5) | < 0.0001 |
