# Supplementary material for: Customised and Noncustomised Birth Weight Centiles and Prediction of Stillbirth and Infant Mortality and Morbidity: A Cohort Study of 979,912 Term Singleton Pregnancies in Scotland
Source: PLoS Med. 2017 Jan 31;14(1):e1002228. doi: 10.1371/journal.pmed.1002228 (PMC5283655; doi:10.1371/journal.pmed.1002228)
Supplement: S2 Table — (DOCX) [file pmed.1002228.s008.docx]

**S2 Table**: Causes of Infant Deaths at term (n = 1,093)

Sudden infant death syndrome (SIDS) was defined as infant deaths for which the primary cause was recorded as an International Classification of Diseases 10 (ICD 10) code of R95 or an ICD 9 code of 798.0. Neonatal causes were coded using a modification of the Wigglesworth paediatric classification, including anoxia and infection.

| Causes of Infant deaths | Number |
| --- | --- |
| SIDS | 394 |
| Anoxia | 270 |
| Intracranial Haemorrhage | 6 |
| Trauma | 7 |
| Infection | 60 |
| Lung related problems | 6 |
| Other haemorrhage | 7 |
| Other paediatric causes | 27 |
| Unexplained (not SIDS) | 19 |
| Unrecorded causes | 297 |
