# Supplementary material for: Customised and Noncustomised Birth Weight Centiles and Prediction of Stillbirth and Infant Mortality and Morbidity: A Cohort Study of 979,912 Term Singleton Pregnancies in Scotland
Source: PLoS Med. 2017 Jan 31;14(1):e1002228. doi: 10.1371/journal.pmed.1002228 (PMC5283655; doi:10.1371/journal.pmed.1002228)
Supplement: S3 Table — (DOCX) [file pmed.1002228.s009.docx]

**S3 Table:** Prediction of stillbirth or infant mortality using customised birthweight or gestation and sex specific birthweight centiles in imputed data.

| **Model** | **AUROC**  **(95% confidence intervals)** | **Improvement in AUROC**  **(P value)** |
| --- | --- | --- |
| **Customised birthweight centile^1^** | 0.61 (0.60 to 0.62) |  |
| **Non-customised birthweight centile^2^** | 0.62 (0.60 to 0.63) | < 0.0001 |

AUROC: Area under the receiver operator characteristic curve

^1^ The birthweight is customised for maternal height, ethnicity (assumed white British), parity, gestation and sex.

^2^: The gestation and sex specific birthweight centile.
