# Supplementary material for: Customised and Noncustomised Birth Weight Centiles and Prediction of Stillbirth and Infant Mortality and Morbidity: A Cohort Study of 979,912 Term Singleton Pregnancies in Scotland
Source: PLoS Med. 2017 Jan 31;14(1):e1002228. doi: 10.1371/journal.pmed.1002228 (PMC5283655; doi:10.1371/journal.pmed.1002228)
Supplement: S4 Table — Analyses undertaken in the ALSPAC cohort (n = 10,378). (DOCX) [file pmed.1002228.s010.docx]

**S4 Table**: Categorisation into small, normal and large for gestational age comparing partial-customisation to the gold-standard of full-customisation using 10^th^ and 90^th^ centile thresholds to define SGA and LGA. Analyses undertaken in the ALSPAC cohort (N = 10,378).

|  | Partial (without maternal weight) Standardisation  Number (% of those with gold standard diagnosis in each category) | | | Total of those with gold standard diagnosis in each category |
| --- | --- | --- | --- | --- |
| Full-customisation (gold standard) | SGA | Normal | LGA |  |
| SGA | 855 (96.6) | 30 (3.4) | 0 | 885 |
| Normal | 182 (2.2) | 7967 (96.8) | 81 (1.0) | 8230 |
| LGA | 0 | 207 (16.4) | 1056 (83.6) | 1263 |
|  |  |  |  | 10,378 |
