# Supplementary material for: Customised and Noncustomised Birth Weight Centiles and Prediction of Stillbirth and Infant Mortality and Morbidity: A Cohort Study of 979,912 Term Singleton Pregnancies in Scotland
Source: PLoS Med. 2017 Jan 31;14(1):e1002228. doi: 10.1371/journal.pmed.1002228 (PMC5283655; doi:10.1371/journal.pmed.1002228)
Supplement: S5 Table — Analyses undertaken in the ALSPAC cohort (n = 10,378). (DOCX) [file pmed.1002228.s011.docx]

**S5 Table**: Categorisation into small, normal and large for gestational age comparing partial-customisation to the gold-standard of full-customisation using 25^th^ and 85^th^ centile thresholds to define SGA and LGA. Analyses undertaken in the ALSPAC cohort (N = 10,378).

|  | Partial (without maternal weight) Standardisation  Number (% of those with gold standard diagnosis in each category) | | | Total of those with gold standard diagnosis in each category |
| --- | --- | --- | --- | --- |
| Full-customisation (gold standard) | SGA | Normal | LGA |  |
| SGA | 2204 (96.5) | 81 (3.5) | 0 | 2285 |
| Normal | 409 (6.5) | 5769 (92.1) | 87 (1.4) | 6265 |
| LGA | 0 | 282 (15.4) | 1546 (84.6) | 1828 |
|  |  |  |  | 10,378 |
