# Supplementary material for: Customised and Noncustomised Birth Weight Centiles and Prediction of Stillbirth and Infant Mortality and Morbidity: A Cohort Study of 979,912 Term Singleton Pregnancies in Scotland
Source: PLoS Med. 2017 Jan 31;14(1):e1002228. doi: 10.1371/journal.pmed.1002228 (PMC5283655; doi:10.1371/journal.pmed.1002228)
Supplement: S1 Text — (DOCX) [file pmed.1002228.s012.docx]

**Supporting Information**

**Methods:**

Data sources

The SMR02 collects information on all women discharged from Scottish maternity hospitals, including maternal and infant characteristics, clinical management and obstetric complications (full lists of data collected are available here: <http://www.adls.ac.uk/nhs-scotland/maternity-inpatient-and-day-case-smr02/?detail>). The SMR02 is subjected to regular quality assurance exercises. The most recent compared a 4.4% sample of SMR02 returns (n=2,531) to case records and demonstrated that all of the data items used in our study were over 90% complete and accurate^1^. Gestational age has been confirmed by ultrasound in the first half of pregnancy in more than 95% of pregnant women in the United Kingdom since the early 1990’s. The NRS (<http://www.nrscotland.gov.uk>) registers all stillbirths and infant deaths and the SSBIDS (http://www.isdscotland.org/Health-Topics/Maternity-and-Births/Stillbirth-and-Infant-Deaths/SPIMMR-Background-Information.pdf) then collects additional information from the SSBIDS coordinator at the relevant hospital; who is an obstetrician, paediatrician or midwife. The coordinator provides copies of the case summaries, post-mortem reports, hospital discharge letters and perinatal mortality meeting reports to the NRS as well as completing a standardised questionnaire that ensures information on the clinical characteristics of the stillbirth are collated for all such deaths. The General Registrar for Scotland collates information from death certificates including the primary and secondary causes of death (<http://www.gro-scotland.gov.uk>).

Calculation of number needed to treat

To estimate the additional number of pregnancies that would need pre-emptive delivery in order to prevent one fatal event, assuming that earlier delivery would be 69% effective at preventing fatal outcomes, compared with the traditional birthweight thresholds of 10^th^ and 90^th^ centiles. We calculated:

(n of additional deliveries with a birthweight ≥10^th^ centile and ≤25^th^ centile)/(n*0.69 of deaths with a birthweight ≥10^th^ centile and ≤25^th^ centile) and (n of additional deliveries with a birthweight ≥85^th^ centile and ≤90^th^ centile)/(n*0.69 of deaths with a birthweight ≥85^th^ centile and ≤90^th^ centile).

Net reclassification index (NRI) formula:

The formula defining the NRI is: NRI=P(up|event)−P(down|event)+P(down|nonevent)−P(up|nonevent)

Where P(up|event) is the proportion of fetuses/infants who experience a fatal event who are placed into a higher risk category with customised centiles compared to non-customised centiles; P(down|event) is the proportion of fetuses/infants who experience an event who are placed into a lower risk category with customised centiles compared to non-customised; P(down|nonevent) is the proportion of fetuses/infants who do not experience an event who are placed into a lower risk category with customised compared with non-customised centiles and P(up|nonevent) the proportion of fetuses/infants who do not experience an event who are placed in a higher risk category with customised compared with non-customised centiles ^2^.

**References**

1. Division IS. Data quality assurance. Assessment of maternity data (SMR02) 2008–2009. *NHS Scotland* 2010.

2. Pencina MJ, D'Agostino RB, Sr., D'Agostino RB, Jr., Vasan RS. Evaluating the added predictive ability of a new marker: from area under the ROC curve to reclassification and beyond. *Stat Med* 2008; **27**(2): 157-72; discussion 207-12.
